# Pollution Status of Pakistan: A Retrospective Review on Heavy Metal Contamination of Water, Soil, and Vegetables

**DOI:** 10.1155/2014/813206

**Published:** 2014-09-03

**Authors:** Amir Waseem, Jahanzaib Arshad, Farhat Iqbal, Ashif Sajjad, Zahid Mehmood, Ghulam Murtaza

**Affiliations:** ^1^Department of Chemistry, Quaid-i-Azam University, Islamabad 45320, Pakistan; ^2^Department of Statistics, University of Balochistan, Quetta 87300, Pakistan; ^3^Institute of Biochemistry, University of Balochistan, Quetta 87300, Pakistan; ^4^COMSATS Institute of Information Technology, Abbottabad 22060, Pakistan

## Abstract

Trace heavy metals, such as arsenic, cadmium, lead, chromium, nickel, and mercury, are important environmental pollutants, particularly in areas with high anthropogenic pressure. In addition to these metals, copper, manganese, iron, and zinc are also important trace micronutrients. The presence of trace heavy metals in the atmosphere, soil, and water can cause serious problems to all organisms, and the ubiquitous bioavailability of these heavy metal can result in bioaccumulation in the food chain which especially can be highly dangerous to human health. This study reviews the heavy metal contamination in several areas of Pakistan over the past few years, particularly to assess the heavy metal contamination in water (ground water, surface water, and waste water), soil, sediments, particulate matter, and vegetables. The listed contaminations affect the drinking water quality, ecological environment, and food chain. Moreover, the toxicity induced by contaminated water, soil, and vegetables poses serious threat to human health.

## 1. Introduction

Those elements required in low quantity by living beings have been identified in the literature as microelements, trace elements, or micronutrients. These elements are necessary for living beings to continue life, but only in minor amount (e.g., vitamins and minerals). Specific nourishing demands of a living body are fulfilled through a combination of all types of macro- and micronutrients [1]. Trace element plays an important role in chemical, biological, biochemical, metabolic, catabolic, and enzymatic reactions in the living cells of plants, animals, and human beings. These elements appear in man and in food and in various environmental compartments in a broad concentration range from natural ultratrace levels at the ppt level and sometimes even below to the often increased due to anthropogenic pollution ppm level [1, 2]. Some of the heavy metals are considered to be xenobiotics because these have no beneficial role in body functioning and are even very harmful in minor concentrations. Cadmium, beryllium, aluminum, uranium, mercury, lead, bismuth, barium, antimony, arsenic, and so forth are included in toxic metals. Higher levels of these metal ions are highly toxic to animals including humans and plants, and their solubility in water is considered to be one of the major environmental issues [3–5]. Environmental challenges of Pakistan are primarily associated with an imbalanced economic and social development in recent decades. All major cities of Pakistan face haphazard, unplanned expansion due to a shift of population from rural to urban areas which worsen the situation to cope up with this challenge. Since the municipal authorities or other utility service providers have limited resources, haphazard urban congestion is the prime reason for deterioration of natural resources (air, water, and soil quality). Access to clean drinking water is limited in developing countries and people may, therefore, consume contaminated water [6, 7]. In Azad Jammu and Kashmir (AJ&K), more than 80% of all illnesses have been attributed to the consumption of poor quality water [8]. It is estimated that water related diseases cause an annual national income loss of Rs. 25–58 billion and over 250,000 children in Pakistan die every year due to diarrheal diseases alone and 20–40% of the hospital beds in Pakistan are occupied by patients suffering from water-related diseases, which are responsible for one-third of all deaths [6, 7]. Only 25.61% (rural 23.5% and 30% urban) of the population in Pakistan have access to safe and drinkable water [7].

Ground water is contributing about one-third in total water resources of Pakistan and is a sole source of water supplies in major municipalities [6, 9]. In Pakistan, main contributors to the surface and ground water pollution are the byproducts of various industries such as textile, metal, dying chemicals, fertilizers, pesticides, cement, petrochemical, energy and power, leather, sugar processing, construction, steel, engineering, food processing, mining, and others [7]. The discharge of industrial effluents, municipal sewage, farm, and urban wastes carried by drains and canals to rivers worsens and broadens water pollution [10, 11].

Sediment analyses play a crucial role in assessing the degree of heavy metal pollution and the resulting health risk associated with the food chain [12]. Coastal areas accommodate well over 60% of Karachi city (Pakistan) industries, including more than 6000 different industrial units such as chemical industries, textiles, pharmaceuticals, metal industries, oil refineries, petrochemical industries, and tanneries [13]. Huge quantities of industrial effluents and domestic sewage discharges, amounting to more than 300 million gallons per day, are directly discharged in the coastal areas via the Lyari and Malir Rivers (Karachi) and many open drains [14]. This, coupled with ever increasing population pressure, urbanization, and industrialization of the coastal areas, not only contributes to huge quantities of effluent discharges but also is of extreme importance as most are unregulated and untreated (less than 20%). Consequently, the coastal marine environment is feared to be exposed to considerable pressure by metal contamination [15]. Disposal of sewage water and industrial wastes is a great problem. Often it is drained to the agricultural lands where it is used for growing crops including vegetables [16, 17]. Wastewater irrigation poses several threats to the environment through contamination by heavy metals. It also poses a number of potential risks to human health via the consumption of or exposure to pathogenic microorganisms and heavy metals [18].

## 2. Pollution Status of Heavy Metal in Pakistan

**Figure 1:**
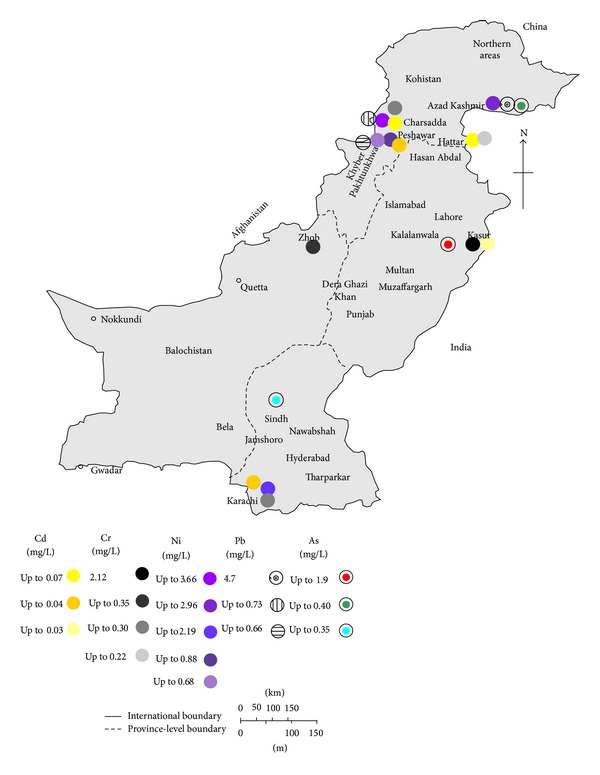
Map of Pakistan showing the Cd, Cr, Ni, Pb, and As concentration in ground water (mean values; where mean value is not available, the highest values are used).

**Figure 2:**
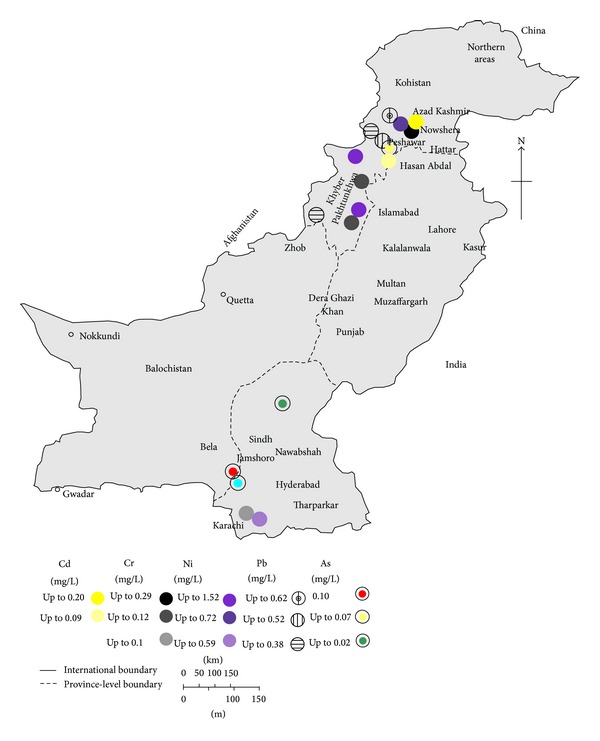
Map of Pakistan showing the Cd, Cr, Ni, Pb, and As concentration in surface water (mean values; where mean value is not available, the highest values are used).

**Figure 3:**
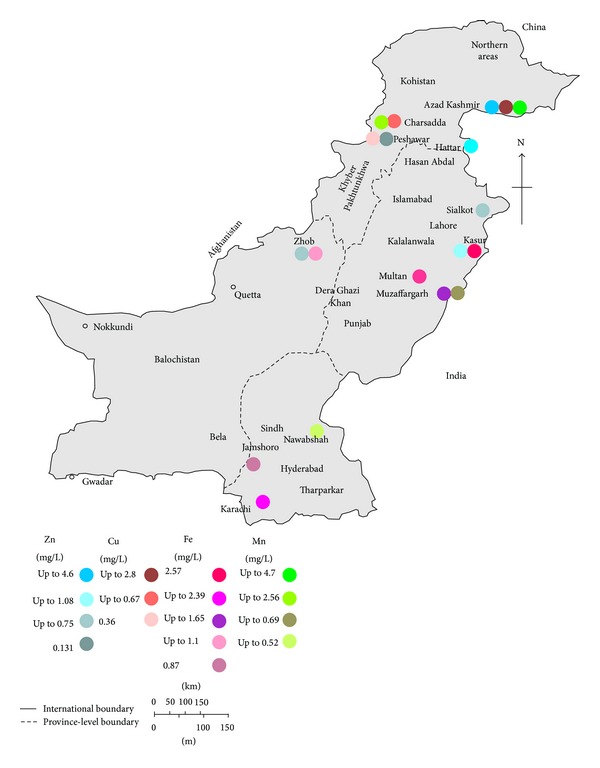
Map of Pakistan showing the Zn, Cu, Fe, and Mn concentration in ground water (mean values; where mean value is not available, the highest values are used).

**Figure 4:**
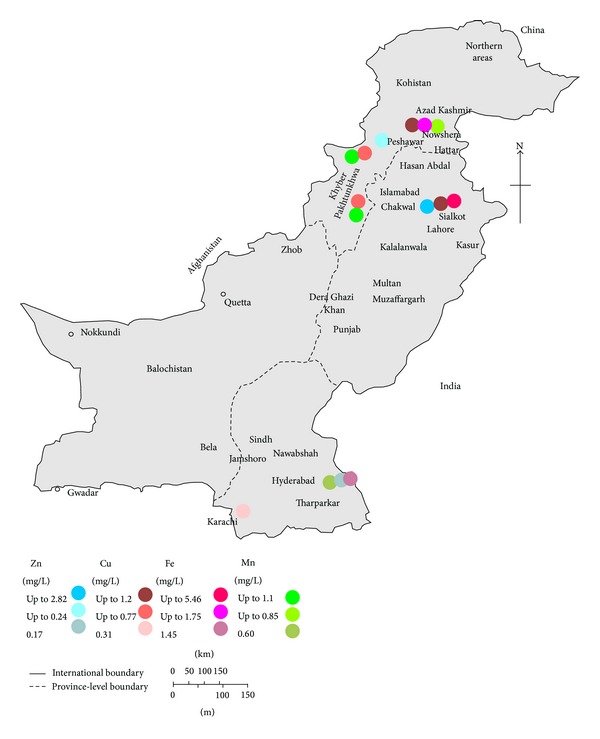
Map of Pakistan showing the Zn, Cu, Fe, and Mn concentration in surface water (mean values; where mean value is not available, the highest values are used).

The environmental and human health effects of heavy metals depend on the mobility of each metal through environmental compartments and the pathways by which metals reach humans and the environment. The pollution status of heavy metals in Pakistan is of great concern and is reflected by the number of studies and accommodated in the form of Tables 1–3, along with maps showing some regions of interest (Figures 1–4). The following paragraph illustrates the current status of heavy metal pollution in different sources like water, soil, sediments, particulate matter, and vegetables.

### 2.1. Arsenic

Arsenic (As) is a toxic element and remains a significant human health concern as As and its compounds (inorganic) are carcinogenic to humans and are classified as Group 1, whereas organic As compounds such as mono- and dimethylarsinic acids are possibly carcinogenic to humans and are classified as Group 2B by International Agency for Research on Cancer [98]. Arsenic exposure causes a markedly elevated risk for developing a number of cancers, most notably skin cancer and cancers of the liver, lung, bladder, and possibly the kidney and colon [99, 100]. During the 1990s, naturally occurring As was found to be widespread in groundwater in the USA, Argentina, Taiwan, China, Hungary, Vietnam, and the Ganges Plain [101]. The World Health Organization provisional guideline value for As concentration in drinking water is 10 *μ*g/L; similarly, various regulatory authorities or countries like USA or EU set guideline values for As due to the growing concern about this poisonous carcinogen andhave raised awareness of the dangers of As in drinking water [20]. Similarly in different areas of Pakistan we are facing the As related severe public health problems as they are present in other neighboring countries and in USA [102–104]. Arsenic concentration was found high in surface and groundwater in Pakistan mainly in two provinces, that is, Punjab and Sindh. Water resources (3% and 16%) having As contamination level of over 50 *μ*g/L are reported in Punjab and Sindh, respectively, while 20% and 36% of water resources of Punjab and Sindh are contaminated with arsenic above 10 *μ*g/L [105, 106]. The Pakistan Council for Research and Water Resources (PCRWR) and UNICEF have undertaken the assessment of drinking water quality since 1999 following the As crisis in Pakistan and other neighboring countries [9]. Consequently, the presence of As contaminated ground waters 10–200 *μ*g/L has been recognized in many areas of Pakistan, especially in Punjab [9, 26]. In 2001, a national survey of arsenic affected drinking groundwater sources was carried out in 35 out of 104 districts in Pakistan [107]. Out of 8712 samples 9% had arsenic concentrations above 10 *μ*g/L guideline value set by the WHO [20] and 0.70% were above 50 *μ*g/L. However, the analysis of 848 samples (out of 8712) shows arsenic concentrations over 10 *μ*g/L in almost 30% of samples and 7% above 50 *μ*g/L.

According to the WHO in developed countries the maximum admissible concentration of As in drinking water is 10 *μ*g/L; however, the developing countries, where arsenicosis is more widespread, are still using the previous guideline value (50 *μ*g/L) due to the lack of facilities to analyze smaller concentrations precisely [20]. The Public Health Engineering Department of Pakistan, in collaboration with UNICEF, conducted a survey for As concentration in drinking water supply wells in 2001 and disclosed some hotspots of As-enriched groundwater occurring in parts of the Indus alluvial basin. Muzaffargarh district was one of the As-enriched hotspots also identified [108]. The same was confirmed later in another study from Muzaffargarh district, which revealed that the As concentration as high as 906 *μ*g/L was present, whereas 58% of samples were found above 10 *μ*g/L [26]. Either direct contamination with industrial or agricultural chemicals or some other anthropogenic influences have been indicated by the spatial distribution of As-rich shallow groundwater [26]. However, in 2007, the enormously high concentrations of As were found in shallow-well waters in four villages from the western and eastern parts of the Punjab, 2400 *μ*g/L in Kalalanwala and Kot Asad Ullah, 883 *μ*g/L in Shamkey Bhatian, 672 *μ*g/L in Manga Mandi, and 681 *μ*g/L in Waran Piran Wala (Table 1, Figure 1) [25]. Arsenic in drinking water causes a widespread concern in southern part of Pakistan, where underground and surface water used for drinking and irrigation are contaminated with As [40]. In the same context, the range of As concentrations in Manchar lake (largest freshwater lake in Pakistan) water of southern part of Sindh was found in the range of 35.2–158 *μ*g/L (mean 97.5 *μ*g/L), which is 3- to 15-folds higher than permissible limit of WHO [40]. Similarly in Jamshoro, Sindh, the highest content of As in surface water sample was found to be 50 *μ*g/L [48].

As concentrations of soil samples are relatively higher in surface soils than in deep soils from the same location. The highest average As content in the soils of the agricultural areas of Sindh (irrigated with As-rich lake water) was found to be 46.2 mg/kg [70] followed by 35 mg/kg in surface soil collected from various regions of Punjab [69]. The author proposed the contribution of air pollutants derived from coal combustion and the use of fertilizers for high levels of As in the surface soils [69]. In some parts of the Sindh total As level in sediment and soil irrigated with lake water was higher than the Threshold Effects Level as reported by Interim sediment quality assessment values [109] and USEPA [110]. The mean values of total As in Manchar lake sediment were found in the range of 11.3–55.8 [70]. Pakistan Environmental Protection Agency reported arsenic in ambient air particulate matter in the Lahore district in the range of 230–2230 ng/m^3^ [87], which is much higher than those reported in the other areas in the world, for example, 91–512 ng/m^3^ in Calcutta, India [111], 25 ng/m^3^ in Wuhan City, China [112], and 1.2–44 ng/m^3^ in Los Angles, USA [113].

Several studies on the linear relationship between As contents of vegetation and concentrations in soils of both total and soluble species suggest that plants take up As passively with the water flow [114]. Plants may accumulate extremely large amounts of As depending on the location and pollution source [114]. It was observed that the use of As-rich irrigation water and soil affected plant height, crop yield, and development of root growth [115, 116]. In south east part of the Sindh the higher accumulation of As was found in spinach, coriander, and mint leaves in the range of 0.90–1.20 mg/kg, while lower uptake of As was observed in onion, carrot, and potato, in the range of 0.048–0.256 mg/kg (Table 3) [70]. The daily As intake from food stuffs in As affected (irrigated with lake water) and unaffected (irrigated with canal water) area was found to be 343.5 and 144.7 *μ*g/day in adults, respectively [70]. This daily dietary intake of total As from food composites by adults is higher than in other countries, 59.2 *μ*g (adult males) and 34.1 *μ*g (adult females) in Canada [117].

### 2.2. Cadmium

Cadmium (Cd) is an element of great concern from toxicity point of view and its exposure can cause both chronic and acute health effects in living organisms. Cd occurs naturally in the earth's crust and in ocean water [98]. The terrestrial abundance of Cd on average is 0.1-0.2 mg/kg, whereas, in ocean waters, it ranges from <5 to 110 ng/L on average [98]. Cd and its compounds are carcinogenic to humans and are classified as Group 1 by International Agency for Research on Cancer, as Cd and its compounds cause cancer of the lung, and positive associations have been observed for cancers of the kidney and of the prostate [98]. Cd intoxication can also leads to pulmonary damages, kidney damage, skeletal damage, and itai-itai diseases [118, 119]. In drinking water the tolerable concentration set by WHO is 0.003 mg/L for Cd [20]. In Pakistan, high Cd concentration in drinking water may be from effluents discharges of marbles, steel, and aluminium industries as well as from mining and metal plating [11]. The observed concentration of Cd in ground water samples collected from various sites of Pakistan ranged from 0.001 to 0.21 mg/L (Table 1) [23, 36]. The highest value of 0.21 mg/L was reported in the samples collected from tube well water of Hayatabad Industrial Estate, Khyber Pakhtunkhwa (KPK) province with an average of 0.02 mg/L [36]. Similarly, the Cd concentration in surface water samples shows large variation throughout the country and ranges between below detection limit to 0.2 mg/L (Figure 2) [53, 56]. Moreover, in surface water sample (Kalar Kahar lake, Chakwal) Cd remained undetectable in the months of March-April; however, it showed seasonal variation in other months of the year (i.e., 0.01–0.05) mg/L [57]. The concentration of Cd in surface water samples collected from various sites in NWFP province (now called KPK) ranged from 0.002 to 0.09 mg/L (mean 0.02 mg/L), the highest value being reported from Kalpani drain. Similarly, Malir River in Karachi (Sindh province) showed variation between 0.002 and 0.07 mg/L Cd (mean 0.04 mg/L) from the same study [10].

A number of studies show the widespread Cd distribution in wastewater samples collected from various regions of Pakistan (Table 1). The highest concentration of 5.35 mg/L Cd in wastewater reported from Korangi area, Karachi [39], exceeded the permissible limit of 0.10 mg/L set by NEQS-Pak for industrial and sewage wastewater [58]. Moreover, in north and east zones of Lahore, Punjab province, the concentration of Cd in wastewater was also above the safe limit set by NEQS and is in the range of 0.18 to 0.37 mg/L [63]. In another study on wetland efficiency for heavy metal removal from industrial wastewater in Gadoon Amazai Industrial Estate, Swabi (KPK province), showed the variation of Cd in the range of 0.19–0.62 mg/L [61].

Natural and anthropogenic sources contribute to the levels of Cd found in soil and sediments, for example, sources like mine/smelter wastes, phosphate fertilizers, or sewage sludge, and municipal waste landfills are the noteworthy [98]. On worldwide level the reported sediment concentrations of Cd range from 0.03 to 1 mg/kg in marine sediments and as high as 5 mg/kg in river and lake sediments ([98] and references therein).

In the soil of various regions of Pakistan, it is observed that there is a large variation in Cd level among the chosen sites, which ranged between 0.02 and 184 mg/kg from normal soil to contaminated soil with mining or other activities (Table 2) [74, 78]. In another study from district Sargodha, the highest concentrations of Cd in the soil was found to be 6.74 mg/Kg and the higher values of Cd in soil suggested the possible risk of Cd entering into higher food chain which was reflected by the Cd accumulation by forage in the range of 1.14 to 4.20 mg/kg [76]. In the soil of Islamabad Territory, capital city of Pakistan, and the dust road along Islamabad Expressway, Cd concentrations of 5.8–6.1 and 4.5–6.8 mg/kg, respectively, have been found; these values are higher than that of many cities around the world, comparable to Aqaba-Shuna Highway (Jordan) and Istanbul Highway (Turkey) [68, 120, 121].

Siddique et al. reported the highest concentration of Cd in sediments, that is, 24.34 mg/Kg at Gizri Creek location at the most downstream part of the Malir River, Karachi, followed by the second highest value 21.34 mg/Kg at the Lyari location, Karachi, where the Lyari River drains the city waste into the Arabian sea [80]. Moreover, in the sediments of the River Ravi, Punjab province, the mean Cd concentrations fluctuated between a maximum value of 3.17 mg/Kg (Shahdera Bridge) and a minimum mean value of 0.99 mg/Kg at Lahore Siphon, Punjab [79]. Toxic metals can enter the human body by consumption of contaminated food crops, water, or inhalation of dust [122]. Various studies from Pakistan suggest the transfer of heavy metals to food crops or vegetables [16, 63, 73, 95, 123]. Critical toxic level of 5.63 mg/kg (average) with reference/control value 2.498 mg/kg of Cd in lettuce irrigated with different levels of wastewater has been reported in Quetta city, Balochistan province [95]. The accumulation of elevated concentration of Cd in lettuce was attributed to the use of wastewater effluents for their cultivation [95]. Cd concentration recorded by different researchers in other parts of the country for various vegetables (including lettuce) clearly indicates the critical toxic level of Cd uptake (Table 3). In Gilgit, Northern Pakistan, the mean concentrations of Cd ranged from 0.24 to 2.1 mg/kg in all vegetable samples, being the highest in* S. oleracea* and the lowest concentration in* M. sylvestris* [73].

Respiration is the one of the two pathways for many metals to enter humans and ingestion with food is the other. Heavy metals in air are a matter of great concern; as we breathe, the polluted air directly transfers the contaminant in to the lungs. Keeping in view of the Cd health impacts, the WHO proposed a guideline value of 5 ng/m^3^ in air [82]. Heavy metals in atmosphere are usually present as a part of fine particles called particulate matter (PM_10_ or PM_2.5_). The IARC Working Group recently classified outdoor air pollution and particulate matter from outdoor air pollution as carcinogenic to humans (IARC Group 1) [124]. The majority of the studies from Pakistan reports the air borne Cd concentration of less than 5 ng/m^3^ (on average basis) in suspended particulate matter (Table 1). However, a report from Lahore shows the annual mean Cd concentration of 69 ng/m^3^ in PM_2.5_ [89].

### 2.3. Lead

Lead (Pb) exposure in children and adults can cause a wide spectrum of health problems, ranging from small effects on metabolism and intelligence to convulsions, coma, renal failure, and death [125]. As per International Agency for Research on Cancer evaluation, inorganic Pb compounds are probably carcinogenic to humans (Group 2A), whereas organic lead compounds are not classifiable as to their carcinogenicity to humans (Group 3) [126]. Lead is found at low concentrations in the earth's crust predominantly as lead sulfide (*galena*), but the widespread occurrence of lead in the environment is largely the result of anthropogenic activity. Pb enters the environment at any stage from its mining to its final use, and it contaminates crops, soil, water, food, air, and dust [126]. In Pakistan, most of the ground water samples exceeded the permissible limit of 0.01 mg/L set by WHO for drinking water (Table 1) and Pb concentration ranges from <0.001 to 4.7 mg/L in various regions (Table 1). Detectable dissolved concentration of Pb in sample collected from Pearl valley of Azad Jammu Kashmir (AJ&K) ranged between 1.8 and 4.7 mg/L [8]. The WHO reference value guidelines comparison has revealed that the concentrations of Pb were 466 higher in water samples taken from the well at Kharick II (South, AJ&K) [8, 20]. In Hattar Industrial Estate (KPK), most of the ground water samples exceeded the critical level of 0.01 mg/L with an average of 0.26 mg/L [36]. Similarly, in Sialkot, Punjab province, 100% of the samples analyzed exceeded the critical level (0.01 mg/L) for lead in drinking water [30]. Individual studies revealed that a higher proportion of water sources in the country had Pb above the safe limits, in both surface and ground waters (Table 1, Figures 1 and 2). In surface waters, significantly higher Pb average concentration has been reported, where the highest value of 0.62 mg/L was observed in Bara River water in Akbarpura area of district Nowshera, KPK [53]. Waste water sample analysis for Pb concentrations from more than 50% studies shows higher values than admissible Pb level of 0.50 mg/L in waste water set by National Environmental Quality Standard Pakistan (Table 1) [58]. The highest Pb contamination (2.34 mg/L) was reported in the samples collected from three textile industries located in Hattar Industrial Estate, KPK [36]. As a consequence, waste water channels have high content of Pb rendering them the most hazardous for soil, plant, and other organisms including human beings.

In soil, Table 2 indicates that the Pb concentration is well below the acceptable level of Pb (50–300 mg/Kg) in normal soil on which sewage sludge is applied by European Union [64]. The only exception in the above statement is where the highest Pb concentration of 103000 mg/kg (mean 1753 mg/kg) was detected in contaminated soil under mining activities with mean reference soil value of 70 mg/kg from Kohistan region, Gilgit Baltistan province [74]. Moreover, the contamination of heavy metals especially Pb in roadside soil is related to the traffic density on the roads [127]. In Pakistan, Pb concentration along National Highway-5 ranges from 12 to 176 mg/kg with a mean of 36.45 mg/Kg and the highest concentration of 176 mg/kg was found near the bypass road of Hyderabad city, Sindh Province, which is the fifth largest industrial city of the country [77].

In various coastal regions of Pakistan, the highest levels of Pb 121 mg/kg were found in the coastal sediments of the Arabian sea along the urban Karachi [80] followed by 49.5 mg/kg from surficial sediments of Lyari River [81].

Lead has been acknowledged as one of the toxic constituents of airborne PM, with emission levels estimated at 450 million kg per annum from industrial coal and oil combustion and 30 million kg per annum from natural sources [114]. The variations in the Pb concentration at some points may be due to traffic burden, brick kilns, and usage of leaded gasoline [68]. Nowadays, the concentration of Pb in the urban atmosphere of Islamabad decreased in recent years due to the use of Pb-free gasoline, although the Pb content is still at a high level, ranging from 0.002 to 4.7 *μ*g/m^3^ [83, 84]. In comparison with the WHO air quality guidelines for Europe (<0.5 *μ*g/m^3^, annual average) [82], the local atmosphere of Islamabad appears to face a serious problem of lead pollution (Table 2). Average air lead levels are usually <0.15 *μ*g/m^3^ at nonurban sites, whereas urban air Pb levels typically range between 0.15 and 0.5 *μ*g/m^3^ in most European cities [82].

According to European Union the permissible level of lead in vegetables are 0.1 to 0.3 mg/kg [90]. In Pakistan, the concentrations of Pb in various vegetable species fluctuating within the range of 0.03–44 mg/Kg [73, 93] and the highest concentration of Pb were observed in* M. sylvestris* from Gilgit (Northern Pakistan) [73]. Another study showed that edible and leafy portions of vegetables had average Pb concentration of 27.49 mg/kg and 15.58 mg/kg, respectively. 83% of the vegetable samples (edible portion) were found well above the EU safe limit [78]. Most of the studies focused on the vegetables grown on contaminated soils due to anthropogenic activities like mining, such as soil amended with sludge or soil treated with wastewater (Table 3).

### 2.4. Nickel

Nickel (Ni) is widely distributed in nature and is found in animals, plants, and soil; the concentration of Ni in soil is approximately in the range of 4–80 ppm [98, 128]. Large amount of Ni is released in the atmosphere due to natural as well as anthropogenic activities including fossil fuel consumption, the industrial production (mining, smelting, and refining), use, and disposal of nickel compounds and alloys, and waste incineration [98]. Human exposure to Ni results from Ni contaminated food ingestion, water, inhalation, and percutaneous absorption [98, 128]. According to International Agency for Research on Cancer evaluation, Ni compounds are carcinogenic to humans and are classified as Group 1. Mixtures of Ni metal and compounds cause cancers of the lung and of the nasal cavity and paranasal sinuses [98]. The maximum permissible concentration for Ni set by WHO in drinking water is 0.07 mg/L [20], whereas National Standards for Drinking Water Quality, Pakistan (NSDWQ-Pak), suggest the guideline value of 0.02 mg/L [19]. The concentration of Ni varies from <0.001–3.66 mg/L in ground water to <0.001–1.52 mg/L in surface water in Pakistan [10]. It was observed that, in most of the cases, groundwater is contaminated with Ni beyond the contamination level set by NSDWQ-Pak or WHO (Table 1). Similarly, 75% of the surface water samples from the largest city of the country (Karachi) exceed the limits [10]. In a study from Lahore (North and East zone), the wastewater samples were collected to evaluate the waste water irrigation impact on vegetables. The Ni concentration was found to be the highest than the reported studies from Pakistan and ranged between 0.91 and 5.94 mg/L and exceeded the permissible limit of 1.0 mg/L set by National Environmental Quality Standards, Pakistan [58, 63].

In soil, the highest concentration of Ni is 324 mg/kg (mean 172 mg/kg) from contaminated Lahore site, while mean reference value of 70 mg/kg (Pb-Zn sulfide horizon/mineralized site) in Kohistan region was found, which is far more than the permissible limits set by EU or USA standards of soil on which sewage sludge can be applied (30–75 and 210 mg/kg), respectively [63, 64]. This was attributed to the dispersion of metals due to mining and may pose potential threats to local communities of Kohistan region. Moreover, in another study conducted on soil of Jhangar Valley, Punjab province, the maximum total content of Ni was recorded as 81 mg/Kg (mean 31.93 mg/kg); the author concluded that these values do not pose any potential health hazard to the general population [72]. In coastal sediments of the Arabian sea along with the urban Karachi, the maximum concentration of 74 mg/kg Ni was found at the Lyari location at the most downstream part of the Malir River [81]. Similarly, in another study the second highest value of 56.46 mg/kg was found at Karachi Port Trust (KPT) Boat Building Area [80]. As per IARC, Ni compounds are human carcinogens by inhalation exposure; therefore, no safe level for nickel compounds can be recommended in air (assuming a linear dose-response) [82, 98]. In the current analysis, the concentration of Ni in particulate matter was reported in the range of 0.001–0.15 *μ*g/m^3^ and the highest of its content was reported in urban atmosphere of Islamabad [85]. In vegetables, the concentrations of Ni ranging from <0.02 to 67.8 mg/kg with mean value of 30.1 mg/kg was observed in vegetables irrigated with sewage water in the suburbs of Peshawar city, KPK [78]. The author reported the significant positive correlation of plant heavy metal with the given heavy metals in soil. In another research, the second highest mean Ni concentration of 28 mg/kg was observed in Spinach (*Spinacia oleracea*) irrigated by sewage water Hasan Abdal area, Punjab province [23].

### 2.5. Copper

Copper is an essential element and is always present in food and in animal liver, which are the major contributors to dietary exposure to copper [129, 130]. Cu acts as a reductant in the enzymes superoxide dismutase, cytochrome oxidase, lysyl oxidase, dopamine hydroxylase, and several other oxidases that reduce molecular oxygen. It is transported in the organism by the protein ceruloplasmin [131]. The recommended dietary allowance (RDA) for adults is 0.9 mg/day. The median dietary intake of copper in USA is approximately 1.0 to 1.6 mg/day and the tolerable upper intake level for adults is 10 mg/day [130]. In Pakistan, surface and ground water contamination with Cu does not pose any significant problems, as most of studies report the Cu concentration within acceptable WHO/NSDWQ-Pak standard limits of 2 mg/L (Table 1, Figures 3 and 4) [20]. There is only one study that shows the detectable dissolved concentration of Cu in ground water ranging from <0.0001 to 2.8 mg/L [8]. Municipal water (well water) from Pothi Bala of AJ&K showed the highest concentration of 2.8 mg/L Cu, while in all other studies the concentration of Cu in drinking water was within the safe limits [8].

According to European Standards, the allowable concentration of Cu in soil (on which sewage sludge is applied) is 50–140 mg/kg (6 < pH < 7) [64, 132]. In various regions of Pakistan, the Cu concentration in soil and dust ranges from <6 to 412 mg/kg (Table 2), where contaminated site from Kohistan region reported the highest content of Cu in soil [74]. The capital city of Pakistan (Islamabad) industrial area shows that the total concentration of Cu is in the range of 8.88–357.40 mg/kg [75]. The highest level of Cu in sediments was found at the Gizri Creek location at the most downstream part of the Malir River, Karachi, 272 mg/kg [80]. Similarly, Rauf et al. reported high level of Cu contents, that is, 159.79 mg/kg, from sediments of River Ravi, Punjab [79]. Higher Cu concentration in soil can cause bioaccumulation in plants, especially the soil which is irrigated by the wastewater or on which sewage sludge is applied. A recent study from Lahore, Punjab province, reported the Cu contents in industrial wastewater irrigated vegetables, compared with the clean reference soil (spinach mean 5.77 mg/kg on contaminated soil and 0.44 mg/kg on clean soil, resp.). The authors concluded that the leafy vegetables have a higher ability to accumulate the heavy metals from soil compared with the other edible parts [63]. In Gillgit, Northern Pakistan, the concentrations of Cu ranged from 09 to 75 mg/kg in all the vegetable samples [73]. These values seem to be alarming keeping in view the tolerable upper intake level of 10 mg Cu/day [130].

### 2.6. Chromium

Chromium is an important element especially in metallurgical/steel or pigment industry. Both of its oxidation forms (+3 and +6) in the chemical are used primarily in pigments, metal finishing, and wood preservatives [114]. The main source of Cr pollution is considered to be from dyestuffs and leather tanning when wastes are discharged directly into waste streams. Cr potentiates the action of insulin and may improve glucose tolerance and its +3 (Cr^3+^, or Cr(III)) form is found in food, which is the most stable oxidation state and its compound occurs naturally [133]. The adequate intake (AI) was established for Cr(III) as 25–35 *μ*g/day (female-male); few serious adverse effects have been associated with excess intake of Cr from food [133]. The toxic form of Cr occurs in +6 oxidation state (Cr(VI)), and its compounds cause cancer of the lung and positive associations have also been observed between exposure to Cr(VI) compounds and cancer of the nose and nasal sinuses [98]. IARC classified Cr(VI) compounds as Group 1 and they are carcinogenic to humans [98]. In ground water samples from various regions in Pakistan show Cr variation ranging from <0.001 to 9.8 mg/L, being the highest (mean value 2.12 mg/L) in well water from residential area, Kasur, Punjab province [27], whereas surface water contamination was found in the range of 0.16–0.29 mg/L Bara River, Nowshera, KPK province (Table 1) [53]. Both of these studies show industrial waste water impacts on water quality. The extent of harm caused by these elevated Cr concentrations in ground and surface water cannot be predicted precisely unless the Cr speciation (Cr(III) or Cr(VI)) is described properly. With a few exceptions [74, 75], most of studies report the Cr content in soil within the acceptable range of 100–150 mg/kg (Table 2), and the world soil average content of Cr has been established as 60 mg/kg [114]. A very high content of Cr in leaf and edible portion of vegetables, that is, 3.74 and 7.56 mg/kg, has been reported [78]. In another study, significantly higher 3.93 mg/kg Cr was reported in spinach irrigated with waste water containing higher Cr content, while irrigation with clean water results 0.004 mg/kg in same vegetable [23].

### 2.7. Iron

Iron is an important element in human body metabolism which acts as a catalyst and is present in greater amount than any other trace element. Iron (Fe) functions as a component of a number of proteins, including enzymes and hemoglobin [130]. The RDA for both male and female is 8 mg/day and the tolerable upper intake level for adults is 45 mg/day of Fe, which is based on gastrointestinal distress as an adverse effect [130]. There is no guideline value sets for Fe in drinking water by WHO, NSDWQ-Pak, or EU standards [19, 20, 64]. Almost all of the studies report the appreciable amount of Fe present in ground and surface water in Pakistan, which helps to keep the required RDA of the population (Table 1). Various studies reported iron concentrations in ground water ranging from <0.01 to 11.8 mg/L and highest concentration reported from Kasur city, Punjab [27], followed by 4.28 mg/L from Jamshoro, Sindh [41]. However, in surface water, the Fe content ranges from 0.01 to 5.46 mg/L in different localities of Pakistan [57]. The permissible value of iron in waste water according to NEQS is 8 mg/L [58]. The analysis of data for waste water in various cities of Pakistan revealed that the most areas have Fe content under the safe limits of 8 mg/L (Table 1) with a few exceptions [36].

Most of the reported studies (Table 2) from different regions of Pakistan reflect the anthropogenic pressure on soil in terms of heavy metal pollution through wastewater/sludge treatment or industrial activities. However, in case of Fe, this pressure buildup does not affect the plant growth as easily soluble and exchangeable fractions of Fe are very low in comparison with the total Fe content in soil [114]. Range of Fe content in soil from different regions is <1 to 196 mg/kg (Table 2). Moreover, exceptionally high value of 25080 to 26960 mg/kg (mean values) of Fe was reported in contaminated soil of Kohistan regions [74]. In current literature survey, the average Fe content in vegetables was found in the range from 7.28 to 500 mg/kg, the highest of all cases found in spinach irrigated by sewage water [23].

### 2.8. Zinc

Zinc is essential micronutrient and catalyzes enzyme activity, contributes to protein structure, and regulates gene expression [130]. Although consequences of Zn deficiency have been recognized for many years but it can be toxic when exposures exceed physiological needs [134]. The adverse effects associated with chronic intake of supplemental Zn include acute gastrointestinal effects and headaches, impaired immune function, changes in lipoprotein and cholesterol levels, reduced copper status, and zinc-iron interactions [133]. The RDA of Zn for adults is 8–11 mg/day (female-male), whereas the tolerable upper intake level is 40 mg/day for adults, a value based on reduction in erythrocyte copper-zinc superoxide dismutase activity [130, 133].

For drinking water NSDWQ-Pak set maximum acceptable concentrations of 5 mg/L for Zn [19]. Both in ground and in surface waters in Pakistan, the Zn level was found well below the standards set by NSDWQ-Pak (Table 1, Figures 3 and 4); however, on the other hand, this data shows the water deficiency in Zn, which hinders this source to meet the RDA. Average zinc (Zn) content of the worldwide soils is estimated to be 70 mg/kg that is the same average level of Zn in the earth's crust [114]. The standard limit of Zn in soil (for sewage sludge applications) set by EU is 150–300 mg/kg [64]. In Pakistan the concentration of Zn in soil/dust varies from >0.1 to 1193 mg/kg (Table 2) with only exception where the highest concentration of Zn in soil/dust was observed in contaminated area, that is, 29755 [64, 74]. However, in road side soil along National Highway (Hyderabad, Sindh province), the Zn varied from 13.8 to 180 mg/kg on dry weight basis [77]. As Zn is another significant element of automobile components, its presence in the roadside soil showed that vehicular traffic is the major anthropogenic source of pollution [77]. Soluble forms of Zn are readily available to plants, and the uptake of Zn has been reported to be linear with metal concentration in the nutrient solution and in soils, and Zn contents of plants vary considerably, reflecting the different factors of various ecosystems and of the genotypes [114]. Background Zn content in lettuce worldwide was found in the range of 44–73 mg/kg [114]. In Pakistan, the highest concentration of Zn of 271 mg/kg was found in* B. campestris* and 247 mg/kg in* M. Sylvestris* vegetables sampled from different parts of Gillgit, Pakistan [73].

## 3. Health Impacts

Heavy metals are of great concern because of their toxic properties and some heavy metals are also essential for the survival and health of humans. However, for these heavy metals (either essential or toxic) the health risk requirement requires consideration of toxicity from excessive exposure. In many parts of the country, heavy metal contamination has been reported as a serious problem but very few studies are available concerning its health effect on the local population. A study conducted by Arain et al. showed that 30–40% people of the Bobak village (near Manchar Lake, Sindh) were suffering from rough skin with black dots and arsenical skin lesions, especially on face, arms, and feet, possibly due to overexposure of high arsenic contents; however, the other factors cannot be ignored [135]. The authors estimated the daily total As intake of 343.5 *μ*g/day for adults from foodstuffs in As affected area (irrigated with lake water), whereas As intake from lake and ground water was estimated in the range of 241–390 *μ*g As/4L/day [135]. Another study revealed that 61 to 73% people of villages on the bank of Manchar Lake suffer from chronic arsenic toxicity like melanosis and keratosis [136]. The authors discovered a strong correlation between arsenic concentrations in drinking water and scalp hair and blood samples of exposed population. Moreover, the exposed people had clinical features like respiratory problems, anemia, gastrointestinal problems, muscles cramps, and weakness. As intake from lake and ground water was estimated in the range of 33.6–390 (males) *μ*g As/4L/day [136]. Similarly, Baig et al. also reported significant correlation between As contents of drinking water and As concentration in scalp hair from subdistrict Gambit (Southern Sindh, Pakistan) [45]. In a comparative cross-sectional study, an association between chronic arsenic exposure through drinking groundwater and decrement in lung function among adult population was reported in Gambat, district Khairpur, Sindh province, in Pakistan [137]. In Pakistan, few studies have been carried out on the health effects of lead through multiple lead exposure sources, although excessively high lead levels in drinking water have been reported in many areas of country (Table 1). One community-based study hypothesized that high lead levels in blood may be a factor associated with hypertension in the Pakistani population [138]. In a study conducted by Rahbar et al., they found that 80% of children of Karachi had elevated blood lead concentrations (>10 *μ*g/dL, with an overall mean of 15.6 *μ*g/dL) due to high level of lead in the air derived from petrol and contamination of food by street dust [139]. Similarly, the mean blood lead levels were found significantly higher in traffic constables from Karachi city (47.7 *μ*g/dL) as compared to Islamabad city (27.2 *μ*g/dL) than control (3.22 *μ*g/dL) [140]. As a consequence, neurological, physiological, and behavioral problems were also observed in exposed population having high level of lead in the blood [139, 140]. Environmental Cd exposure in schoolchildren of the Lahore region has been reported recently [141]. In the same cross-sectional study, the Cd association was reported with bone resorption, suggesting a direct osteotoxic effect with increased calciuria [141].

## Figures and Tables

**Table 1:** Heavy metal contamination in fresh and waste water.

S. number	Sample type and location (water)	**Zn (mg/L) range** **(mean)**	Cu (mg/L) range (mean)	Fe (mg/L) range (mean)	Mn (mg/L) range (mean)	Cd (mg/L) range (mean)	Cr (mg/L) range (mean)	Ni (mg/L) range (mean)	Pb (mg/L) range (mean)	As (mg/L) range (mean)	Ref.
A	**Ground water**										
	**NSDWQ-Pak (drinking water)**	5.0	2.0	—	0.5	0.01	0.05	0.02	0.05	0.05	[19]
	**WHO (drinking water)**	—	2.0	—	—	0.003	0.05	0.07	0.01	0.01	[20]
1	Zhob River basin, Balochistan. Open & deep well water	0.03–0.75	0.07–0.25	0.5–1.1	0.06–0.25	—	0.06–0.35	—	0.02–0.06	—	[21]
2	Kalalanwala area near Lahore, Punjab (shallow tube wells *n* = 17)	—	—	—	—	—	—	—	—	0.02–1.889	[22]
3	Tube well water, Hasan Abdal, Punjab	(0.009)	(0.02)	(0.07)	(0.02)	(0.001)	(0.04)	(0.03)	(0.03)	—	[23]
4	Kalalanwala, Punjab (*n* = 24)	—	—	—	—	—	—	—	—	0.032–1.90	[24]
5	Well water, villages near Kalalanwala, Punjab (*n* = 123)	—	—	—	—	—	—	—	—	0.001–2.40 (123)	[25]
6	Well water, Multan district, Punjab (*n* = 3)	—	—	0.07–2.7	0.06–1.0	—	—	—	—	0.06–1.0	[26]
	Well water, district Muzaffargarh, Punjab (*n* = 49)			0.0–1.65	0.0–0.69					0.0–0.40	
7	Well water from residential area, Kasur, Punjab (*n* = 68)	0.01–1.08	—	0.02–11.8 (2.57)	0.01–0.17	0.001–0.027	0.05–9.80 (2.12)	0.001–0.24	0.003–0.26	—	[27]
8	Eight districts of Punjab (*n* = 4547)	—	—	—	—	—	—	—	—	0.006–0.012 (0.0085)	[28]
9	Dera Ghazi Khan, Punjab (*n* = 32)	—	—	—	—	—	—	—	—	0.001–0.029	[29]
10	Sialkot, Punjab (*n* = 25)	0–0.81 (0.16)	0.01–0.17 (0.06)	0–0.83 (0.30)	0–0.09 (0.03)	—	0–0.30 (0.03)	0.01–0.22 (0.10)	0.11–0.81 (0.49)	—	[30]
11	Urban areas of Lahore, Punjab	—	—	0.01–2.32 (0.77)	—	—	—	—	0–0.007 (0.002)	0.024–0.0716 (0.036)	[31]
12	Sunken tube-wells, Kahna, Lahore, Punjab	—	—	—	—	—	—	—	—	(0.071)	[32]
13	Shallow wells in the vicinity of Palosi, Peshawar, KPK (*n* = 13)	0.047–0.34	0.26–0.34	0.0–0.30	0.071–0.21	0.0–0.04	—	0.0–0.68	0.27–0.38	—	[33]
	Deep wells in the vicinity of Palosi drain Peshawar (*n* = 3)	0.0–0.082	0.558–0.598	0.46–0.99	0.0–0.306	0.0–0.056	—	0.0–0.52	0.0–0.49	—	
14	Well water, Charsadda and Risalpur, KPK (*n* = 8)	0.002–0.27	0.004–0.67	0.01–0.43	0.08–2.56	0.01–0.07	0.01–0.30	0.002–3.66	0.02–0.73	—	[10]
	Well water, Korangi, Karachi, Sindh (*n* = 8)	0.04–0.52	0.01–0.21	0.51–2.39	0.07–0.12	0.02–0.04	0.003–0.07	0.01–2.19	0.10–0.24		
15	Tap water, Industrial Estate, Hattar, KPK	0.050	0.030	0.50	0.15	—	—	—	0.001	—	[34]
16	Tube well and dug well, Hayatabad Industrial Estate, Peshawar, KPK	(0.10)	(0.36)	(0.08)	(0.12)	(0.03)	(0.09)	(0.88)	(0.66)	—	[11]
17	Water near tannery effluent (*n* = 38), Peshawar, KPK	0.001–0.559 (0.131)	—	0.003–1.097 (0.143)	0.001–0.740 (0.087)	0.003–0.043 (0.014)	0.012–0.145 (0.089)	0.004–0.454 (0.136)	0.004–0.407 (0.080)	—	[35]
18	Hattar Industrial Estate, KPK (*n* = 90)	0.007–1.340 (0.18)	—	0.005–1.070 (0.110)	0.004–0.18 (0.04)	0.001–0.21 (0.02)	0.010–0.940 (0.22)	0.009–0.585 (0.08)	0.001–2.34 (0.26)	—	[36]
19	Drinking water, Peshawar industrial area, KPK, Polluted area	—	—	—	—	—	—	—	—	0.004–0.059	[37]
	Control area									0.009–0.038	
20	Hand pump, Khairpur/Matiari, Sindh (*n* = 16)	—	—	—	—	—	—	—	—	(0.25)	[38]
21	Tube well water, Korangi, Karachi, Sindh (*n* = 4)	(0.048)	(0.032)	(2.39)	(0.124)	(0.041)	(0.3)	(0.656)	(0.24)	—	[39]
22	Hand pumps water in the vicinity of Manchar lake, Sindh (*n* = 1944)	—	—	—	—	—	—	—	—	0.0233–0.096	[40]
23	Jamshoro, Sindh (*n* = 309)	—	—	0.09–4.28 (0.79)	—	—	—	—	—	0.0013–0.106 (0.040)	[41]
24	Talukadaur, district Nawabshah, Sindh (*n* = 38)	0–0.228	0–0.099	0.075–1.35	0.001–0.517	0.002–0.017	—	0.013–0.09	0.006–0.053	—	[42]
25	Industrial area, Karachi, Sindh	—	(0.049)	—	—	—	—	—	—	—	[43]
26	Sindh (*n* = 240)	—	—	—	—	—	—	—	—	0.0087–0.352	[44]
27	Hyderabad city, Sindh	—	—	—	—	—	—	—	—	0.0045–0.010 (0.0087)	[45]
	Tharimirwa, Sindh									0.014–0.057 (0.0284)	
	Gambat, Sindh									0.0388–0.362 (0.112)	
28	Tap water, Karachi, Sindh	—	(0.121)	(0.106)	(0.081)	—	(0.012)	(0.037)	(0.006)	—	[46]
29	18 districts of Karachi, Sindh (*n* = 108)	—	—	—	—	—	—	—	(0.146)	—	[47]
30	District Jamshoro, Sindh	—	—	0.09–4.3 (0.87)	—	—	—	—	—	0.013–0.106 (0.040)	[48]
31	Diplo and Chachro subdistrict of Tharparkar, Sindh	—	—	0.1–0.32	—	—	—	—	—	0.0001–0.002 (0.0011)	[49]
32	Mithi and Nagarparkar subdistrict of Tharparkar, Sindh	—	—	0.076–0.27	—	—	—	—	—	0.0001–0.003 (0.0011)	[50]
33	Drinking water, Pearl valley, Azad Jammu and Kashmir (18 locations)	<0.009–4.6	<0.0001–2.8	—	<0.002–4.7	—	—	<0.002–2.96	<0.03–4.7	—	[8]
34	Kohistan region, Northern Pakistan (*n* = 37)	—	—	(0.28–0.42)	—	—	—	—	—	(0.0001–0.006)	[51]
35	Kohistan region, Northern Pakistan	—	—	—	—	—	—	—	—	—	
	Jijal-dubair area (*n* = 10)	0.029–2.881 (0.651)	0.035–0.133 (0.738)		0.023–0.009 (0.005)	0–0.001 (0.001)	0.002–0.035 (0.021)	0–0.014 (0.005)	0–0.005 (0.002)		[52]
	Besham (*n* = 15)	0.165–3.387 (1.376)	0.015–0.102 (0.042)		0.002–0.066 (0.019)	0–0.004 (0.002)	0–0.004 (0.001)	0–0.006 (0.002)	0.001–0.043 (0.002)		
	Alpuri (*n* = 12)	0.032–2.414 (0.827)	0.033–0.043 (0.0391)		0.002–0.025 (0.012)	0–0.0004	0–0.0022 (0.001)	0.002–0.010 (0.005)	0.002–0.003 (0.002)		

B	**Surface water**										
1	Kabul River, Peshawar, KPK	—	(0.07)	(1.1)	—	(0.03)	—	(0.2)	(0.52)	—	[17]
2	Palosi drain, Peshawar, KPK (*n* = 4)	0.0–0.239	—	0.37–0.75	0.017–0.24	0.0–0.004	—	0.0–0.18	0.0–0.34	—	[33]
3	Surface water from different spots in KPK (*n* = 16)	0.003–0.08	0.01–0.77	0.01–1.29	0.01–1.11	0.002–0.09	0.01–0.12	0.01–1.52	0.02–0.38	—	[10]
	Malir River, Karachi, Sindh (*n* = 8)	0.06–0.29 (0.16)	0.01–0.84 (0.31)	0.13–2.91 (0.78)	0.05–0.57 (0.33)	0.002–0.07 (0.04)	0.03–0.29 (0.10)	0.02–1.06 (0.59)	0.09–0.32 (0.19)		
4	Peshawar Industrial area, KPK	—	—	—	—	—	—	—	—	0.016–0.071	
	Polluted area									0.0395–0.038	[37]
	Control area										
5	Bara River, Nowshera, KPK (*n* = 9)	0.02–0.06	0.90–1.20	1.29–1.75	0.77–0.85	0.15–0.20	0.16–0.29	0.53–0.72	0.43–0.62	—	[53]
6	Warsak dam, KPK	(0.087)	(0.042)	—	—	—	(0.051)	(0.012)	(0.009)	—	[54]
7	Phulali canal, Hyderabad, Sindh (*n* = 6)	(0.167)	(0.063)	(1.451)	(0.596)	(0.004)	(0.0082)	(0.005)	(0.026)	—	[55]
8	Manchar lake, Jamshoro, Sindh (*n* = 9)	(0.016)	(0.009)	(0.012)	—	(0.001)	—	(0.004)	(0.009)	—	[56]
9	Manchar lake, Jamshoro, Sindh (*n* = 540)	—	—	—	—	—	—	—	—	0.0035–0.157 (0.097)	[40]
10	Jamshoro, Sindh (*n* = 309)	—	—	0.08–0.38 (0.19)	—	—	—	—	—	0.003–0.037 (0.015)	[41]
11	Sindh (*n* = 480)	—	—	—	—	—	—	—	—	0.003–0.018	[44]
12	18 Districts of Karachi, Sindh (*n* = 108)	—	—	—	—	—	—	—	(0.077)	—	[47]
13	District Jamshoro, Sindh	—	—	0.02–0.38 (0.16)	—	—	—	—	—	0.003–0.050 (0.015)	[48]
14	Kalarkahar lake, Chakwal, Punjab	0.44–2.82	0.01–1.20	0.20–5.46	—	0.01–0.05	—	0.04–0.25	0.01–0.30	—	[57]

C	**Waste water**										
	**NEQS-Pak**	5.0	1.0	8.0	1.5	0.1	1.0	1.0	0.5	1.0	[58]
1	City and industrial effluent at Budni nala, Peshawar, KPK	—	(0.5)	(3.5)	—	(0.5)	(1.8)	(1.5)	(1.3)	—	[17]
2	Textile effluents in the Kabul River, KPK	—	0.04–0.17	—	0.01–0.1	0–0.16	0.5–0.8	0–0.04	0.07–0.14	—	[59]
3	Palosi drain Peshawar, KPK	(0.23)	—	0.37–0.75 (0.63)	0.02–0.24	0–0.03	—	(0.14)	0–0.34	—	[33]
4	Textile industries, Hattar Industrial Estate, KPK (*n* = 90)	0.014–2.482 (0.26)	—	0.039–30.3 (2.14)	0.011–0.97 (0.16)	0.004–0.181 (0.04)	0.023–2.671 (0.71)	0.013–2.482 (0.26)	0.013–2.34 (0.28)	—	[36]
5	Korangi area, Karachi, Sindh (*n* = 24)	0.005–5.5	0.005–1.19	0.04–5.58	0.01–1.79	0.004–2.4	0.004–5.62	0.02–5.35	0.05–2.25	—	[39]
6	City sewage, Hattar Industrial Estate, KPK	(0.21)	(1.2)	(2.46)	(0.15)	—	—	(0.32)	(0.002)	—	[34]
7	Hayatabad Industrial Estate, Peshawar, KPK	(0.01)	(0.36)	(0.42)	(0.16)	(0.04)	(0.06)	(1.25)	(0.70)	—	[35]
8	Industrial Estates of Pakistan				—	—	—	—			[60]
	Peshawar, KPK (*n* = 3)	(6.0)	(0.59)	(2.22)					(0.309)	(0.643)	
	Gujranwala, Punjab (*n* = 3)	(9.0)	(0.61)	(2.03)					(0.35)	(0.475)	
	Hattar, KPK (*n* = 3)	(8.0)	(0.40)	(3.80)					(0.27)	(0.942)	
9	Effluent industrial area, Amangarh Nowshera, KPK	0.75–0.59	15–0.62	—	—	—	0.66–0.03	0.01–1.45	0.11–0.07	—	[54]
10	Gadoon Amazai Industrial Estate, Swabi, KPK, wastewater treatment wetland (*n* = 3)	—	0.75–1.45	0.07–0.27	—	0.19–0.62	0.07–0.35	1.63–2.76	0.78–1.56	—	[61]
11	Hudiara drain, Lahore, Punjab	(1.7)	(0.45)	7–9	(0.85)	(0.18)	(0.07)	(0.93)	(0.03)	—	[62]
12	Hasan Abdal, Punjab	(0.42)	(0.70)	(2.67)	(0.53)	(0.031)	(0.71)	(2.23)	(0.42)	—	[23]
13	North and East zone of Lahore, Punjab (urban and industrial wastewater)	0.34–1.39	9.28–32.69	—	0.19–1.13	0.18–0.37	0.19–0.54	0.91–5.94	0.26–0.70	—	[63]

**Table 2:** Heavy metal contamination in soil, sediments, and particulate matter.

	Sample type and location	Zn (mg/kg) range (mean)	Cu (mg/kg) range (mean)	Fe (mg/kg) range (mean)	Mn (mg/kg) range (mean)	Cd (mg/kg) range (mean)	Cr (mg/kg) range (mean)	Ni (mg/kg) range (mean)	Pb (mg/kg) range (mean)	As (mg/kg) range (mean)	Ref.
A	** Soil/dust**										
	**EU (**6 < pH < 7**)**	**150–300**	**50–140**	—	—	**1–3**	**100–150**	**30–75**	**50–300**	—	[64]
	**USA**	**1400**	**750**	—	—	**20**	**1500**	**210**	**150**	—	
	**(soil on which sewage sludge is applied)**										
1	Soil, Hattar Industrial Estate, KPK (*n* = 90)	0.02–49.64 (2.61)	—	0.78–196 (15.02)	0.02–7 (1.21)	0.02–1.4 (0.17)	0.240–34.06 (5.96)	0.02–18.4 (1.51)	0.02–23.02 (4.46)	—	[65]
2	Soil near tannery effluent, Peshawar, KPK (*n* = 38)	0.23–8.42 (2.381)	—	0.11–87.10 (18.03)	0.43–9.68 (5.29)	0.14–1.0 (0.60)	0.81–100.2 (29.9)	1.02–9.72 (4.45)	2.27–8.43 (4.66)	—	[66]
3	Soil amended with sewage sludge, Hyderabad, Sindh (*n* = 6) (wet acid digestion method)	(208.6 ± 6.5)	(32.2 ± 1.52)	—	—	(4.3 ± 0.41)	(34.6 ± 2.1)	(6.2 ± 0.48)	(67.4 ± 4.0)	—	[67]
4	Soil from Islamabad capital territory (*n* = 4)	69.20–115 (91.05)	20–30 (25)	—	—	5.8–6.1 (5.95)	—	30–38 (32)	40–90 (62.5)	—	[68]
	Islamabad expressway dust	64.3–169 (116)	30–80 (52)			4.5–6.8 (5.0)		10–30 (23)	60–150 (104)		
5	Soil, Punjab (*n* = 45)	—	—	—	—	—	—	—	—	7–35 (11.08)	[69]
6	Soil from South East of Sindh (*n* = 200)	—	—	—	—	—	—	—	—	8.7–46.2	[70]
7	Soil along motorway (M-2) (3 regions and 2 zones) (*n* = 397)	(81.40)	—	—	—	(0.46)	(14.61)	(14.19)	(0.5)	—	[71]
8	Soil, Jhanjar valley, Punjab (*n* = 14)	56–136 (97.5)	—	—	—	—	12–67 (34.29)	15–81 (31.93)	3–24 (14.86)	—	[72]
9	Soil, Gilgit area, North Pakistan (*n* = 11)	—	—	—	—	—	**—**	**—**	**—**	—	
	Kondas	(590)	(147)				(1.0)	(52)	(35)		[73]
	Dainyor	(1193)	(99)				(0.85)	(31)	(36)		
	Nagirl	(172)	(55)				(0.3)	(36)	(43)		
	Jageer Baseen	(210)	(72)				(0.75)	(57)	(29)		
	Naltar	(460)	(71)				(2.3)	(24)	(138)		
10	Soil, industrial zone of Islamabad	61.9–172.6	8.9–357.4	—	—	—	[74]	41.4–59.3	2.0–29.0	**—**	[75]
11	Soil at livestock experimental station, Khizerabad, district Sargodha, Punjab	—	—	—	—	—	0.006-0.007	—	—	—	[76]
12	Soil along the N-5 national highway near various cities (Lahore, Multan, Bahawalpur, Rahim Yar Khan, Ubauro, Sukkur, Moro, Hyderabad, Karachi) (*n* = 27)	13.83–180 (56.72)	5.26–26.88 (12.98)	2.70–7.98 (3.96)	117.6–309 (174.09)	0.56–1.25 (0.84)	—	5.96–13.23 (8.82)	12.3–176 (36.46)	—	[77]
13	Soil, Pb–Zn sulfide horizon/mineralized site, Kohistan region, North Pakistan	— —	— —	— —	— —	— —	— —	— —	— —	—	[74]
	Contaminated Pazang site	95–1072 (361)	31–412 (193)	12715–63815 (25080)	465–5735 (2437)	0.6–5.0 (2.0)	42–756 (146)	64–318 (99)	12–1337 (117)		
	Reference Pazang site	24–179 (115)	23–114 (68)	1200–23225 (14645)	14–702 (471)	0.4–3.2 (1.3)	60–409 (252)	51–253 (158)	23–81 (51)		
	Contaminated Lahore site	213–29755 (5123)	111–345 (205)	17250–51750 (26960)	266–15420 (5779)	0.4–184 (20)	92–850 (439)	18–324 (172)	5–10300 (1753)		
	Reference Lahore site	86–410 (202)	25–79 (48)	5840–13905 (10958)	269–1048 (636)	1.8–3.4 (2.8)	123–154 (139)	26–48 (35)	23–97 (70)		
14	Soil collected from 40 different localities around Peshawar, KPK	—	—	—	—	0.02–0.37 (0.24)	0.04–1.92 (0.35)	0.02–1.11 (0.65)	0.02–7.52 (2.58)	—	[78]

	**Sediments**										
1	Coastal area, Karachi, Sindh										
	Buleji	(0.101)	(0.010)	(5.064)	(5.550)	(0.007)	(0.040)	(0.034)	(0.088)		[15]
	Paradise point	(0.035)	(0.013)	(7.410)	(4.950)	(0.014)	(0.027)	(0.038)	(0.070)		
	Nathia Gali	(0.041)	(0.013)	(7.790)	(5.060)	(0.006)	(0.024)	(0.030)	(0.059)		
2	Manchar lake, Sindh (*n* = 200)	—	—	—	—	—	—	—	—	11.3–55.8	[70]
3	River Ravi, Punjab (*n* = 19)	—	3.38–159.79	—	—	0.99–3.17	4.60–57.40	—	—	—	[79]
4	Coastal sediments of the Arabian sea along the urban Karachi, Sindh (9 locations) (*n* = 88)	3.31–389.23	62.95–272	0.55–6.47 (3.51)	318–1.15	0.01–24.34	2.95–180.90	7.48–74.91	6.33–121.03	—	[80]
5	Surface sediments in Karachi coastal sites, Sindh	15.6–666 (204.79)	—	—	300–1300 (500)	—	12.0–319 (96.75)	1.53–58.9 (31.39)	9.0–49.5 (23.24)	—	[81]
	**Particulate matter (*μ*g/m** ^ 3^ **)**										
	**WHO**	—	—	—	0.15	0.005	—	—	0.5	—	[82]
1	Islamabad, Station 1 (*n* = 44) and Station 2 (*n* = 61)	—	—	—	—	—	—	—	0.004–4.07 (4.07) 0.016–4.0 (3.98)	—	[83]
2	Islamabad, urban site (*n* = 96) and rural site (*n* = 79)	0.003–2.350 (0.567) 0.011–1.790 (0.666)	—	0.037–2.950 (0.667) 0.002–1.990 (0.573)	0.011–0.314 (0.056) 0.002–0.400 (0.066)	0.002–0.007 (0.003) 0.001–0.017 (0.002)	0.001–0.398 (0.036) 0.001–0.042 (0.008)	0.001–0.064 (0.008) 0.001–0.065 (0.004)	0.003–4.0 (0.163) 0.002–4.075 (0.327)		[84]
3	Urban areas of Islamabad (*n* = 181)	0.02–2.07 (1.021)	—	0.03–5.97 (1.761)	0.008–0.08 (0.055)	0.001–0.01 (0.004)	0.002–0.08 (0.018)	0.006–0.15 (0.017)	0.013–0.36 (0.128)	—	[85]
4	Urban atmosphere, Islamabad (*n* = 209)	0.022–8.62 (1.702)	—	0.24–6.218 (2.073)	0.005–0.37 (0.073)	0.001–0.019 (0.004)	0.002–0.08 (0.018)	0.002–0.08 (0.018)	0.002–0.89 (0.182)	—	[86]
5	Ambient air particulate matter, Lahore, Punjab	—	—	—	—	—	—	—	—	0.23–2.23	[87]
6	Particulate Matter in urban atmosphere of Islamabad (*n* = 214)	0.022–13.2 (2.311)	0.023–7.51 (0.306)	0.088–11.2 (2.464)	0.005–0.52 (0.079)	0.001–0.019 (0.004)	0.001–0.08 (0.010)	0.002–0.13 (0.024)	0.005–0.89 (0.144)	—	[88]
7	Atmosphere of Lahore, Punjab	(10)	(0.012)	(1.7)	(0.44)	(0.069)	(0.012)	(0.0044)	(3.6)	—	[89]
	PM_2.5_	(11)	(0.003)	(8.2)	(2.1)	(0.07)	(0.0014)	(0.018)	(4.4)		
	PM_10_										

**Table 3:** Heavy metal contamination in vegetables.

	Sample type and location (vegetables)	Zn (mg/kg) range (mean)	Cu (mg/kg) range (mean)	Fe (mg/kg) range (mean)	Mn (mg/kg) range and (mean)	Cd (mg/kg) range (mean)	Cr (mg/kg) range (mean)	Ni (mg/kg) range (mean)	Pb (mg/kg) range (mean)	As (mg/kg) range (mean)	Ref.
1	**EU**	—	—	—	—	0.05–0.2	—	—	0.1–0.3	—	[90]

2	Spinach (*Spinacia oleracea*)	—	—	—	—	—	—	—	—	—	
	Irrigated by sewage water	(26.169)	(28.56)	(500)	(104.10)	(3.20)	(3.93)	(28.0)	(10.43)		
	Irrigated by sewage + tube well water	(16.38)	(26.66)	(430)	(73.26)	(0.35)	(2.14)	(7.1)	(4.10)		
	Irrigated by tube well water	(7.71)	(8.30)	(24.57)	(15.30)	(0.005)	(0.004)	(0.13)	(0.16)		[23]
	Okra (*Abelmoschus esculentus*)	—	—	—	—	—	—	—	—		
	Irrigated by sewage water	(20.05)	(22.91)	(353.8)	(104.10)	(2.60)	(3.09)	(20.8)	(9.35)		
	Irrigated by sewage + tube well water	(11.53)	(20.45)	(300.12)	(73.26)	(0.36)	(1.77)	(6.03)	(2.13)		
	Irrigated by tube well water	(4.26)	(6.32)	(16.221)	(15.30)	(0.003)	(0.004)	(0.11)	(0.18)		

3	*Withania somnifera* collected from different locations of KPK province	—	— —	— —	— —	— —	— —	— —	— —	—	[91]
	Root		0.26–0.32	10.9–13.5	1.67–3.5	—	0.03–0.07	0.05–0.08	0.06–0.16		
	Stems		0.36–0.38	7.28–12.9	1.52–2.42	0.01–0.06	0.02–0.05	0.02–0.04	0.04–0.46		
	Leaves		0.28–0.40	14.6–19.74	1.52–6.27	0.02	0.06–0.10	0.05–0.17	0.10–0.23		

4	Gilgit, Northern Pakistan (*n* = 5)	—	—	—	—	—		—	—	—	
	*M. sylvestris *	(247)	(20)			(0.24)		(10)	(20)		[73]
	*B. campestris *	(271)	(17)			(0.62)		(04)	(17)		
	*S. oleracea *	(40)	(11)			(2.10)		(07)	(18)		
	*M. sylvestris *	(78)	(28)			(0.67)		(20)	(44)		
	*C. intybus *	(240)	(48)			(0.81)		(12)	(42)		
	*T. repens *	(50)	(15)			(0.86)		(12)	(18)		
	*A. viridis *	(50)	(17)			(0.79)		(11)	(16)		
	*P. oleracea *	(96)	(09)			(0.94)		(10)	(16)		
	*B. oleracea *	(115)	(17)			(0.72)		(24)	(35)		
	*L. sativa *	(54)	(24)			(0.84)		(11)	(15)		
	*M. neglecta *	(07)	(75)			(1.55)		(15)	(10)		

5	Industrial area, Faisalabad, Punjab	—	—	—	—	—	—	—	—	—	
	Leaves	—	—			—	—		—		[92]
	Spinach	(0.461)	(0.923)			(0.035)	(0.217)		(2.251)		
	Coriander	(0.705)	(0.653)			(0.062)	(0.369)		(2.652)		
	Lettuce	(0.743)	(0.851)			(0.049)	(0.434)		(2.411)		
	Radish	(1.893)	(0.273)			(0.033)	(0.288)		(2.035)		
	Cabbage	(0.777)	(0.252)			(0.073)	(0.336)		(1.921)		
	Cauliflower	(0.678)	(0.323)			(0.064)	(0.546)		(1.331)		
	Stem	—	—			—	—		—		
	Spinach	(0.361)	(0.529)			(0.061)	(0.492)		(1.193)		
	Coriander	(0.874)	(0.855)			(0.026)	(0.376)		(1.642)		
	Lettuce	(0.498)	(0.751)			(0.015)	(0.495)		(1.883)		
	Radish	(0.813)	(0.346)			(0.017)	(0.386)		(2.161)		
	Cabbage	(0.539)	(0.161)			(0.023)	(0.426)		(1.624)		
	Cauliflower	(0.456)	(0.207)			(0.025)	(0.386)		(1.313)		
	Roots	—	—			—	—		—		
	Spinach	(0.781)	(0.391)			(0.052)	(0.511)		(1.121)		
	Coriander	(0.745)	(0.931)			(0.026)	(0.502)		(1.531)		
	Lettuce	(1.637)	(0.352)			(0.015)	(0.511)		(1.854)		
	Radish	(0.813)	(0.462)			(0.051)	(0.489)		(2.254)		
	Cabbage	(0.442)	(0.361)			(0.013)	(0.543)		(1.152)		
	Cauliflower	(0.564)	(0.221)			(0.011)	(0.338)		(1.222)		

6	South East part of Sindh	—	—	—	—	—	—	—	—	—	
	Irrigated with lake water									—	
	Bitter gourd									(0.811)	[70]
	Brinjal									(0.570)	
	Carrot									(0.135)	
	Coriander									(0.985)	
	Okra									(0.894)	
	Onion									(0.048)	
	Potatoes									(0.256)	
	Sponge gourd									(0.504)	
	Spinach									(0.90)	
	Peppermint									(1.20)	
	Irrigated with canal water									—	
	Bitter gourd									(0.275)	
	Brinjal									(0.350)	
	Carrot									(0.058)	
	Coriander									(0.185)	
	Okra									(0.324)	
	Onion									(0.018)	
	Potatoes									(0.0565)	
	Sponge gourd									(0.187)	
	Spinach									(0.187)	
	Peppermint									(0.450)	

7	Sindh, Pakistan (*n* = 210)	—	—	—	—	—	—	—	—	—	[93]
	Leafy vegetables (coriander, methi, spinach, and mint)					(0.083) —			(0.050) —	(0.042) —	
	Tuberous vegetables (arum, onion, potato, radish, sugar beet, turnip)					(0.057) —			(0.03) —	(0.045) —	
	Cucurbit vegetables (bitter ground, cucumber, Indian squash, pumpkin)					(0.021) —			(0.051) —	(0.056) —	
	Fruity vegetables (brinjal, cabbage, cauliflower, chilies, French bean, okra, tomato)					(0.035)			(0.067)	(0.054)	

8	Vegetables from Faiz Ganj, Punjab	—	—	—	—	—	—	—	—	—	[94]
	Okra									(0.20)	
	Sponge gourd									(0.360)	
	Brinjal									(0.170)	
	Bittle gourd									(0.275)	
	Bottle gourd									(0.390)	
	Cluster beans									(0.603)	
	Spinach									(0.280)	
	Peppermint									(1.01)	
	Indian squash									(0.804)	
	Peas									(0.630)	
	Vegetables from Thari Mirwah									—	
	Okra									(0.80)	
	Sponge gourd									(0.504)	
	Brinjal									(0.390)	
	Bittle gourd									(0.811)	
	Bottle gourd									(1.05)	
	Cluster beans									(0.734)	
	Spinach									(0.90)	
	Peppermint									(1.20)	
	Indian squash									(1.30)	
	Peas									(0.910)	
	Vegetables from Gambat									—	
	Okra									(0.890)	
	Sponge Gourd									(0.612)	
	Brinjal									(0.570)	
	Bittle Gourd									(1.11)	
	Bottle Gourd									(1.25)	
	Cluster beans									(1.30)	
	Spinach									(1.10)	
	Peppermint									(1.70)	
	Indian squash									(1.63)	
	Peas									(1.03)	

9	Lettuce (*Lactuca sativa* L.) collected from three different localities of Quetta, Balochistan	48.5–94.37 (76.457)	—	—	21.38–32.46 (26.11)	2.49–7.45 (5.63)	—	0.76–3.59 (3.08)	1.85–8.58 (5.94)	—	[95]

10	Local and important vegetable									—	[96]
	Onion	(37.6)	(4.8)	(47.4)	(8.24)	(0.6)	(1.13)	(0.89)	(3.04)		
	Potato	(37.66)	(6.7)	(44.3)	(5.27)	(0.66)	(2.1)	(0.59)	(2.2)		

11	Vegetables irrigated with sewage water, Peshawar, KPK	—	—	—	—	— —	— —	0.4–56 (30.78)	— —	—	[78]
	Leaves of 40 different vegetables					(12.37)	(3.74)	0.4–67.8	(15.58)		
	Edible portion of 40 different vegetables					(27.67)	(7.56)	(30.14)	(27.49)		

12	Vegetables collected from local market of Shorkot, Sindh	0.245–3.873	0.109–0.401	3.45–41.28	0.220–3.334	0.002–0.627	0.211–1.298	0.117–1.190	0.046–1.159	—	[97]

13	Vegetables irrigated with waste water, Lahore, Punjab	— —	— —	—	— —	— —	— —	— —	— —	—	[63]
	Potato	10.35–42.8 (21.77)	1.09–6.12 (4.16)		10.43–30.91 (17.77)	0.08–0.35 (0.21)	0.88–2.61 (1.60)	1.29–19.02 (6.04)	0.19–0.63 (0.49)		
	Cabbage	21.98–53.91 (37.76)	1.63–7.8 (4.92)		9.29–30.2 (17.26)	0.17–0.36 (0.27)	2–4.019 (3.06)	0.96–14.67 (5.046)	1.88–4.23 (2.86)		
	Cauliflower	20.98–55.91 (35.44)	2.1–6.09 (4.49)		6.57–11.18 (9.06)	0.18–0.31 (0.23)	1.15–4.15 (2.15)	0.76–8.91 (2.95)	0.91–3.07 (1.66)		
	Brassica	35.43–63.91 (47.10)	1.84–10.17 (5.42)		57.84–81.01 (69.9)	0.32–1.73 (1.08)	1.37–4.87 (2.62)	1.00–21.91 (6.43)	1.12–4.03 (2.77)		
	Turnip	21.17–35.25 (28.53)	2.87–6.5 (4.90)		14.47–25.83 (19.5)	0.20–0.67 (0.36)	0.29–3.02 (1.44)	1.02–32.51 (5.76)	0.19–1.29 (0.77)		
	Spinach	18.18–31.26 (25.86)	2.14–10.94 (5.77)		11.83–21.73 (16.58)	1.20–3.08 (1.90)	1.37–4.27 (2.42)	0.18–11.1 (3.65)	1.88–4.19 (2.9)		
	Beet	34.73–60.26 (45.65)	1.9–6.49 (4.1)		67.39–87.37 (76.26)	019–0.23 (0.21)	0.29–2.61 (1.31)	1.1–31.03 (6.23)	0.21–0.93 (0.64)		
	Garlic	19.71–29.05 (24.64)	1.17–8.35 (4.70)		16.84–27.09 (21)	0.09–0.27 (0.19)	3.18–5.01 (4.1)	0.87–20.67 (9.85)	0.71–0.31 (0.38)		
	Carrot	14.04–25.95 (19.39)	1.9–9.51 (5.34)		11.34–22.85 (17.45)	0.11–0.57 (0.29)	0.98–3.01 (1.92)	1.13–9.97 (5.33)	1.01–1.97 (1.97)		
	Coriander	33.83–48.26 (39.69)	1.57–4.94 (4.95)		— (28)	0.13–0.61 (0.31)	1.23–4.37 (2.32)	1.09–15.57 (6.2)	1.64–2.53 (2.04)		
